# Aortic arch endovascular branch and fenestrated repair: Initial Canadian experience with novel technology

**DOI:** 10.1016/j.jvscit.2023.101274

**Published:** 2023-07-27

**Authors:** Mark Rockley, Kenton L. Rommens, R. Scott McClure, Eric J. Herget, Holly N. Smith, Randy D. Moore

**Affiliations:** a Division of Vascular Surgery, Department of Surgery, University of Calgary, Calgary, AB, Canada; b Division of Cardiac Surgery, Department of Cardiac Sciences, University of Calgary, Calgary, AB, Canada; c Division of Interventional Radiology, Department of Radiology, University of Calgary, Calgary, AB, Canada

**Keywords:** Aortic aneurysm, Aortic dissection, Cardiac surgery, TEVAR, Vascular surgery

## Abstract

**Objective:**

The objective is to describe the initial Canadian experience using novel aortic arch branched endograft technologies.

**Methods:**

We performed a retrospective consecutive case series of all patients undergoing aortic arch branched repair with newly available endograft technology since 2020 at our site. We describe the patient characteristics, treatment characteristics, and postoperative outcomes.

**Results:**

Eleven patients received arch branched endografts, indicated for penetrating aortic ulcer in seven patients (64%), arch degeneration after prior aortic dissection repair in three (27%), and acute aortobronchial fistula in one patient (9%). Their average age was 72 ± 7 years. Complete arch repair from zone 0 to 4 was performed in six cases (55%); the remaining repairs landed proximally in zones 1 or 2. Seven repairs used a single retrograde facing inner branch (thoracic branch endoprosthesis; W.L. Gore & Associates), three used double antegrade inner branch (Bolton Relay; Terumo Interventional Systems), and one emergent case used double in situ fenestrations. Seven repairs (64%) used an adjunctive extra-anatomic bypass to complete great vessel perfusion, two of which were created during a prior aortic repair. Inferior vena cava balloon inflow occlusion during deployment was used in all cases. No mortalities, transient or permanent spinal cord paralysis, myocardial infarction, dialysis dependence, venous thromboembolism, or bleeding requiring reintervention occurred. No patient undergoing elective arch branch repair experienced a stroke. The one patient undergoing emergent repair did suffer a stroke. The median length of stay was 5 days (interquartile range, 2-8 days). Two endoleaks developed: a type Ia endoleak successfully treated with a Palmaz stent (Cordis) during the index admission, and a type II endoleak with ongoing sac regression on postoperative follow-up. Postoperatively, one patient suffered a suspected aortic graft infection that was treated with lifelong antibiotics. During a mean radiographic follow-up of 7.2 months, no cases of branch vessel instability (ie, no migration, reintervention, arterial rupture, intraluminal thrombus, occlusion, stenosis, or kinking of the branch grafts) developed. Three patients experienced sac regression of >5 mm, and no patient experienced continued postoperative dilation.

**Conclusions:**

To the best of our knowledge, this is the largest reported Canadian volume of aortic arch repair using novel branched or fenestrated technology. The series demonstrates that a multidisciplinary program and properly selected patients can yield excellent results using endovascular repair for complex aortic arch pathology.

Open surgery is the established gold standard to treat pathology of the aortic arch,^1^ with an estimated operative mortality and permanent stroke rate of 5.3% and 3.4%, respectively.^2^ However, in high-risk patient cohorts, mortality has been reported to approach 7%.^3^ Total arch replacement is a maximally invasive surgical procedure, and patients remain highly selected owing to both the mortality risk and the inherent morbidity of this approach.^3^ In response to the anatomic and physiologic challenges of open aortic arch repair, hybrid techniques have been developed that combine open great vessel debranching and standard thoracic endovascular stent repair (TEVAR).^4^ Hybrid techniques avoid or minimize the complexity of open aortic surgery to mitigate the perioperative morbidity relative to total arch replacement. Although these hybrid alternatives can prove beneficial for select patients, to date, the results have been mixed.^4^, ^5^, ^6^, ^7^

Total endovascular aortic arch repair (TEAAR) has emerged as a potential alternative to treat aortic arch pathology in nonoperative or high-risk surgical patients, showing high intraprocedure success and an acceptable outcome profile.^8^^,^^9^ Flow can be maintained to the great vessels via custom-made branch devices,^10^^,^^11^ off-the-shelf branch devices,^12^ or in situ fenestrations.^13^, ^14^, ^15^ Branched or fenestrated aortic arch devices can be arranged with single, double, or triple branches.^16^ TEAAR has allowed patients with less favorable comorbidity profiles to be considered for aortic arch repair.^8^ Compared with hybrid repair, purely endovascular aortic arch branched repair is associated with shorter operative times and lengths of stay, without increases in complication rates at mid-term follow-up.^17^ In conjunction with TEAAR devices for zone 0 deployment, endograft deployment in zone 2 with branch extension into the left subclavian artery, negating the need for carotid–subclavian bypass in anatomically suitable patients, has further advanced the use of endovascular therapies at the distal arch.^17^ This is broadly applicable beyond high-risk patients and complements current TEVAR indications.

Our center advocates and uses a multidisciplinary approach to complex aortic disease, consisting of cardiac and vascular surgeons and interventional radiologists working together. This breadth of skill set in this collaboration allows clinicians to tailor the aortic repair approach to suit individual patient characteristics. Our approach has encouraged several device manufacturers to pilot new devices at our institution before dissemination to the broader Canadian market. Thus, we have accrued a reasonable volume of endovascular aortic arch repairs within a short period. The purpose of our report is to describe the patient selection, treatment characteristics, and outcomes of patients undergoing endovascular aortic arch branched or fenestrated repair at our center.

## Methods

We performed a retrospective single-center consecutive case series of patients undergoing aortic arch repair with fenestrated or branched endografts between December 2020 and March 2023. All the patients received a multidisciplinary consultation before proceeding with branched arch endograft placement. A branched aortic arch endograft was defined as an aortic stent graft extending proximal to the left subclavian artery (zone 2) and landing distal to the left subclavian artery (zone 3+),^18^ with a minimum of one fenestration or branched stent extending into a great vessel distal to the most proximal extent of the aortic graft fabric. Further extra-anatomic debranching of a vessel perfused by the branched great vessel was included. Any device manufacturer with custom-made devices, in situ fenestrations, and parallel stent grafts was eligible for inclusion. Both percutaneous and open surgical exposure of access vessels were eligible. Elective and emergent cases were included. The indications for intervention include aneurysmal disease and acute aortic syndrome. Regarding penetrating aortic ulcer (PAU), our indications to repair a PAU with an arch branch stent are any attributable symptoms, an absolute diameter of 40 mm, a width of 20 mm, and/or a depth of 10 mm.^19^ In the absence of these indications, we serially image PAUs using annual computed tomography angiography (CTA).

The recorded variables were categorized into patient characteristics, treatment characteristics, clinical surgical outcomes, and technical surgical outcomes. The patient characteristics included age at the procedure, urgency of intervention, etiology and anatomic extent of pathology according to the Society for Vascular Surgery clinical practice guidelines,^18^ cardiovascular surgical history, and comorbidities using the Elixhauser comorbidity index.^20^ The treatment characteristics included technique of arch endovascular debranching, endovascular devices used, number of great vessel branches, vascular access sites, cardiac output suppression strategy, spinal drain usage, and operative duration. The clinical surgical outcomes included retrograde aortic dissection, length of stay, occurrence of transient ischemic attack, stroke, myocardial infarction, cardiac valvular dysfunction, transient or permanent spinal cord ischemia, renal failure, superficial wound or graft infection, and venous thromboembolism, and aortic-related and all-cause mortality. We also documented the postoperative antiplatelet and anticoagulant plan.

The technical surgical outcomes included intraoperative or postoperative endoleak, secondary reinterventions, and primary, assisted primary, and secondary branch patency. We also recorded the target vessel instability components, a composite outcome that included any target vessel complication leading to death, arterial rupture, occlusion, component separation, or endoleaks.

We leveraged a province-wide electronic health records system (ConnectCare EPIC) to ensure complete follow-up of all patients within the catchment area. No unnecessarily identifying information was collected to protect patient confidentiality. Because of the necessity of capturing a consecutive case series, the requirement for patient informed consent was waived. The local ethics board approved the present study, and our study is reported in line with the PROCESS (preferred reporting of case series in surgery) guidelines.^21^

## Results

Eleven patients met the inclusion criteria for aortic branched endografts during the study period of 28 months. The average age at surgery was 72 ± 7 years. One case was performed emergently for aortobronchial fistula; the remaining patients were asymptomatic and underwent elective surgery for de novo PAU (45%), arch degeneration after prior aortic dissection repair (36%), or an intramural hematoma (9%). Overall, 45% of the patients had a history of symptomatic acute aortic syndrome, and 55% had undergone prior aortic surgery. The underlying comorbidities are listed in Table I.

**Table I:** Preoperative patient characteristics

Characteristic	Value
Patients	11 (100)
Age, years	72 ± 7
Male sex	6 (55)
Emergent	1 (9)
Indication	
De novo penetrating aortic ulcer	5 (45)
Arch degeneration after prior aortic dissection repair	4 (36)
Intramural hematoma	1 (9)
Aortobronchial fistula	1 (9)
Prior aortic dissection	5 (45)
Prior aortic surgery	6 (55)
Smoking	6 (55)
COPD	2 (18)
Atrial fibrillation	4 (36)
Hypertension	10 (91)
Dyslipidemia	6 (55)
Diabetes	1 (9)
Chronic renal failure	2 (18)
Dialysis dependence	0 (0)
Stroke	2 (18)
Transient ischemic attack	1 (9)
Coronary artery disease	4 (36)

*COPD,* Chronic obstructive pulmonary disease.

Data presented as number (%) or mean ± standard deviation.

Six of the repairs (55%) involved total arch endografting, which landed in zone 0 proximally (Table II). Zone 1 was the site of two additional repairs, and zone 2 was the proximal site for the remaining three. Seven of the repairs (64%) used a single retrograde facing inner branch arrangement (thoracic branch endoprosthesis; W.L. Gore & Associates), three used a double antegrade inner branch arrangement (Bolton Relay custom-made device; Terumo Interventional Systems), and one emergent case used two in situ branch fenestrations to the innominate and left carotid arteries. Of the 16 branch stents used, 7 were balloon-expandable stents (Viabahn VBX; W.L. Gore & Associates), and 9 were self-expanding covered stents (thoracic branch endoprosthesis; Viabahn; Excluder iliac limb extension; W.L. Gore & Associates). All retrograde facing branch stent extensions were self-expanding. Seven of the 11 repairs (64%) used an adjunctive extra-anatomic bypass or transposition to complete great vessel perfusion, two of which had already been performed for a prior aortic repair. All percutaneous axillary access sites used 5F sheaths and were closed with Angio-Seal (Terumo Interventional Systems). Inferior vena cava (IVC) balloon occlusion (Coda LP balloon, Cook Medical) was used for cardiac suppression during main body deployment in all cases by inflation within the right atrium, followed by tension into the atriocaval junction to occlude the IVC.

**Table II:** Aortic branch stent characteristics

Pt. No.	Branched aortic device	Proximal–distal landing zone	Length of aortic coverage, mm	Branch vessels	Branch arrangement	Branch stent device (n)	Extra-anatomic debranching
1	Bolton Relay	0-4	150	IA, LCA	Antegrade inner branch	VBX (2)	LCSB (remote)
2	TBE	2-5	150	LSCA	Retrograde inner branch	TBE branch (1)	–
3	TBE	1-5	150	LSCA	Retrograde inner branch	TBE branch (1)	CCT
4	TBE	2-5	228	LSCA	Retrograde inner branch	TBE branch (1)	–
5	TBE	0-5	385	IA	Retrograde inner branch	TBE branch (1)	CCT and LCSB
6	Bolton Relay	0-4	120	IA, LCA	Antegrade inner branch	VBX (2)	LCSB (remote)
7	Bolton Relay	0-4	60	IA, LCA	Antegrade inner branch	VBX (1), Excluder limb (1)	–
8	RIBS (cTAG)	0-4	200	IA, LCA	In situ fenestration	VBX (2)	LCSB
9	TBE	1-5	280	LSCA	Retrograde inner branch	Viabahn (1)	LCST
10	TBE	0-4	172	IA	Retrograde inner branch	TBE branch (1)	CCT and LCSB
11	TBE	2-4	150	LSCA	Retrograde inner branch	TBE branch (1)	–

*CCT,* Left to right carotid–carotid transposition; *IA,* innominate artery; *LCA,* left carotid artery; *LCSB,* left carotid–subclavian bypass; *LCST,* left carotid-to-subclavian transposition; *LSCA,* left subclavian artery; *Pt. No.,* patient number; *RIBS,* retrograde in situ branch stenting; *TBE,* thoracic branch endoprosthesis.

The mean operative time was 4.3 ± 1.9 hours, with a wide range related to the surgical complexity (Table III). Spinal drains were used in all procedures with either coverage below T8 (27%) or prior distal thoracoabdominal aortic repair, and the average length of aortic coverage was 186 mm. No iatrogenic arterial dissections or aortic valvular injuries were noted during intraoperative transesophageal echocardiography. One patient did suffer an intraoperative iliac artery rupture secondary to a 26F sheath, which was treated successfully intraoperatively with covered iliac stenting.

**Table III:** Operative conduct

Pt. No.	Femoral access	Great vessel access	Largest sheath, F	Cardiac output suppression	Operative time, minutes	Spinal drain placed
1	Open, 1 femoral	Open bilateral carotid	26	IVC occlusion	325	No
2	Percutaneous, 1 femoral	Percutaneous left axillary	26	IVC occlusion	364	No
3	Percutaneous, 1 femoral	Percutaneous left axillary	26	IVC occlusion	167	No
4	Percutaneous, 1 femoral	Percutaneous left axillary	24	IVC occlusion	135	Yes
5	Percutaneous, 2 femoral	Percutaneous right axillary	24	IVC occlusion	274	Yes
6	Open, 1 femoral; percutaneous, 1 femoral	Open bilateral carotid arteries	22	IVC occlusion	284	No
7	Percutaneous, 2 femoral	Open left carotid and right axillary	24	IVC occlusion	230	No
8	Percutaneous, 2 femoral	Percutaneous left axillary, open bilateral carotid	22	IVC occlusion	493	No
9	Percutaneous, 2 femoral	Open left axillary	26	IVC occlusion	185	Yes
10	Percutaneous, 2 femoral	Percutaneous right axillary	26	IVC occlusion	250	No
11	Percutaneous, 1 femoral	Percutaneous left axillary	22	IVC occlusion	107	No

*IVC,* Inferior vena cava; *Pt. No.,* patient number.

During inpatient recovery, six patients went to the intensive care unit, most commonly for spinal cord drain management (Table IV). The maximum intensive care unit stay was 3 days, and the median total hospital length of stay was 5 days (interquartile range, 2-8 days). No transient or permanent spinal cord ischemia complications, myocardial infarctions, renal failure requiring dialysis, venous thromboembolism, or bleeding requiring reintervention occurred. One patient developed pneumonia during inpatient recovery and was successfully treated with oxygen therapy and antibiotics. The patient with an aortobronchial fistula who received an emergent total arch repair with in situ fenestration suffered bilateral infarcts in a central embolic pattern on postoperative day 1, without stent graft pathology such as branch kinking or thrombus. CTA after the stroke demonstrated a right-sided carotid cutdown arteriotomy site focal stenosis at the site of prior purse-string closure, which was patch repaired on postoperative day 5 to maximize cerebral perfusion. After prolonged rehabilitation, this patient made a meaningful recovery and was ambulating independently on discharge.

**Table IV:** Perioperative outcomes

Pt. No.	ICU, days	Total LOS, days	SCI	CVA	MI	Valvular dysfunction	Dialysis	Pneumonia	Infection	Bleeding	Postoperative antiplatelet	Postoperative anticoagulant
1	0	8	No	No	No	No	No	No	No	No	No	DOAC
2	0	3	No	No	No	No	No	No	No	No	DAPT × 3 months	No
3	0	1	No	No	No	No	No	No	No	No	No	DOAC
4	2	5	No	No	No	No	No	No	No	No	DAPT × 3 months	No
5	2	2	No	No	No	No	No	No	No	No	Lifelong DAPT	No
6	2	6	No	No	No	No	No	No	Yes	No	No	DOAC
7	2	11	No	No	No	No	No	Yes	No	No	DAPT × 3 months	No
8	3	58	No	Yes	No	No	No	No	No	No	Lifelong aspirin	DOAC
9	2	5	No	No	No	No	No	No	No	No	Lifelong DAPT	No
10	0	6	No	No	No	No	No	No	No	No	Lifelong DAPT	No
11	0	1	No	No	No	No	No	No	No	No	DAPT × 3 months	No

*CVA,* Cerebrovascular attack (transient or permanent); *DAPT,* dual antiplatelet therapy; *DOAC,* direct oral anticoagulant; *LOS,* length of stay; *MI,* myocardial infarction; *Pt. No.,* patient number; *SCI,* spinal cord ischemia (transient or permanent).

All patients were discharged with either dual antiplatelet or anticoagulation therapy, if otherwise medically indicated. All patients have recorded at least one postoperative outpatient clinical contact. The average total follow-up in our series is 7.8 months. One month postoperatively and after discharge, patient 6 was diagnosed with a presumed aortic arch graft infection based on clinical and nuclear medicine findings, despite persistently negative blood cultures. The total aortic arch repair with a branched stent graft was performed for dissection-related aortic degeneration, after remote acute type A dissection ascending aortic repair and subsequent descending aortic TEVAR. The patient was ultimately treated with lifelong broad-spectrum antibiotics, with radiographic nuclear imaging improvement of infection after 7 months of antibiotics.

All 11 patients underwent completion angiography intraoperatively and postoperative CTA before discharge, with an outpatient surveillance CTA plan at 3 and 6 months and annually thereafter at a minimum. Three patients experienced sac regression of >5 mm, and no patient experienced continued postoperative dilation (Table V). Two patients developed an endoleak. The first patient had a persistent type Ia endoleak identified on routine inpatient postoperative CTA requiring Palmaz stenting (Cordis) during the same admission 1 week after the index aortic arch stent placement, with resolution of endoleak on subsequent imaging. The other endoleak was type II endoleak from an intercostal artery, which was monitored and showed ongoing sac regression. No patient developed branch vessel instability; no branches developed migration, occlusion, reintervention, arterial rupture, intraluminal thrombus, stenosis, or kinking. No patient in this series died.

**Table V:** Postoperative follow-up

Pt. No.	Endoleak	Aortic sac regression, mm	TVI	In-hospital mortality	Follow-up mortality	Follow-up, months
1	Type Ia, treated successfully with Palmaz stent	−7	No	No	No	27
2	No	−3	No	No	No	12
3	No	−1	No	No	No	12
4	No	−2	No	No	No	8
5	Type II, observed	−13	No	No	No	8
6	No	−1	No	No	No	8
7	No	0	No	No	No	5
8	No	−11	No	No	No	3
9	No	−1	No	No	No	1
10	No	0	No	No	No	1
11	No	−2	No	No	No	1

*Pt. No.,* Patient number; *TVI,* target vessel instability (composite outcome of any target vessel complication leading to death, arterial rupture, occlusion, component separation, or endoleak).

## Discussion

This case series highlights the added value of using aortic arch branched endografts. The comorbidities and prior aortic surgeries of our study population reflect previous reports of high-risk open arch repair.^3^ Despite this, the procedures were well tolerated, with a median hospital admission of 5 days. The indication for repair for most patients in this series was a focal PAU or aortic arch degeneration with prior surgical repair of the ascending and descending thoracic aorta. Most cases in this series were performed exclusively percutaneously to further minimize the operative burden; open vascular access was typically only required for redo femoral exposure or retrograde placement of large caliber stents through carotid or axillary access. No spinal cord complications occurred, reflecting both preservation of the distal thoracic aorta in most cases and maintenance of left subclavian perfusion in all but one patient with limited thoracic aortic coverage (patient 7).

In addition, no branch vessel complications developed. Our practice is generally to use self-expanding branch grafts when advanced from femoral access, taking advantage of the lower profile balloon-expandable grafts when advancing retrograde from great vessel access. In the absence of a medical indication for anticoagulation, our practice is to prescribe dual antiplatelet therapy (aspirin and clopidogrel [Plavix; Bristol-Myers Squibb – Sanofi Pharmaceuticals partnership]) for a minimum of 3 months, followed by lifelong aspirin, with lifelong dual antiplatelet therapy for patients with zone 0 or zone 1 deployment. Although every patient received dual antiplatelet or anticoagulant therapy, only one endoleak (type II) was seen on the most recent postoperative imaging study of all patients in this series. This might reflect the ability of aortic arch branch technology to move the landing zone of a TEVAR proximally into a confident length of normal ascending or arch aorta without significant additional morbidity. The absence of endoleaks could also represent the paucity of small branch vessels that contribute to type II endoleaks after endovascular repair of the more distal aorta. The short postoperative recovery and acceptable complication rate demonstrate that a well-selected, yet relatively comorbid, population can tolerate aortic branch repair.

To facilitate a zone 1 landing of an aortic endograft with a single branch into the left subclavian artery with adequate proximal landing zone, we performed a left carotid-to-subclavian transposition. This “reverse” transposition avoids a prosthetic graft and is markedly simpler than the standard left subclavian-to-carotid transposition, because the subclavian artery needs only to be exposed distally to the vertebral origin, and the common carotid artery is easily mobilized. These factors lead to less hazardous subclavian dissection and clamping. In particular, a left carotid-to-subclavian transposition is useful for a zone 1 landing site when the distance between the innominate and left carotid arteries are too short to accommodate a left carotid artery branch.

This series also includes an emergent aortic arch retrograde in situ branch stenting using a recently reported gutter balloon technique to maintain cerebral perfusion for an aortobronchial fistula.^13^ However, this patient also experienced the only stroke in our series. The precise mechanism of this event remains unclear, because the patient did have bilateral infarcts in a central embolic pattern. This case also emphasizes the potential value of readily accessible “off-the-shelf” aortic branch devices in the future.

Finally, this case series demonstrates the benefits of a collaborative interdisciplinary aortic team. We have experienced accelerated adoption of aortic arch branched repair since the program's inception, with wide industry support for this model of service delivery, leading to enhanced access of novel approved technology. Our group has trialed alternative cardiac suppression, including pharmacologic therapy, cardiac pacing, and Valsalva maneuver; however, we have selected IVC occlusion as the most reliable method. Patient selection is critical for successful aortic branch repair. Accordingly, clinical success due to proper patient selection relies on the program's ability to offer a multitude of treatment options. These nuanced decisions require a breadth of expertise in both the clinic and the operating room. This case series underscores the value of a multidisciplinary aortic program to rapidly adopt and implement complex aortic repair.

Endovascular arch branch technology is an emerging field, and although this case series contributes to existing literature, long-term data with more volume and robust trials free from selection bias are required to confidently evaluate the durability of these repairs and the standing of endovascular arch repair in relationship to open repair.

## Conclusions

To the best of our knowledge, this is the largest reported Canadian volume of aortic arch branched repair using novel branched or fenestrated technology. This series demonstrates that a multidisciplinary program and properly selected patients can yield excellent results using endovascular repair for complex aortic arch pathology.
